# Supplementary material for: Comparative analysis of chloroplast genomes indicated different origin for Indian tea (Camellia assamica cv TV1) as compared to Chinese tea
Source: Sci Rep. 2021 Jan 8;11:110. doi: 10.1038/s41598-020-80431-w (PMC7794434; doi:10.1038/s41598-020-80431-w)
Supplement: Supplementary file 1 — Supplementary Information. [file 41598_2020_80431_MOESM1_ESM.docx]

**Supplementary Data**

**Comparative analysis of chloroplast genomes indicated different origin for Indian Tea (*Camellia assamica* cv TV-1) as compared to Chinese tea**

Hukam C. Rawal^1^, Sangeeta Borchetia^2^, Biswajit Bera^3^, S. Soundararajan^3^, R Victor J Ilango^4^, Anoop Kumar Barooah^2^, Tilak Raj Sharma^1^, Nagendra Kumar Singh^1^, Tapan Kumar Mondal^1^*

^1^ICAR-National Institute for Plant Biotechnology, LBS Centre, IARI, New Delhi, India.

^2^Tocklai Tea Research Institute, Tea Research Association, Jorhat, Assam, 785008, India.

^3^Tea Board, Ministry of Commerce and Industry, Govt. of India, 14, B.T.M. Sarani, Kolkata, 700 001, India.

^4^UPASI Tea Research Foundation, Tea Research Institute, Coimbatore, Tamil Nadu, India.

*Corresponding author


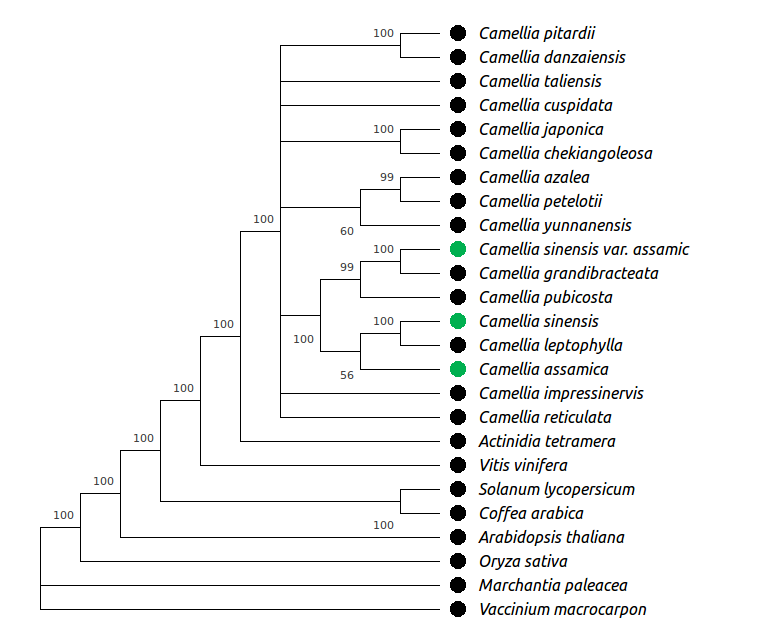


Supplementary Fig. S1. UPGMA method based phylogenetic tree between 7 cp genome sequences of Camellia species. Sequences were aligned with ClustalW and Evolutionary analyses were conducted in MEGA X at 1000 bootstrap support.

Table S1. List of chloroplast (cp) genome sequence used in the analysis

| **S. No.** | **Accession** | **Species** | **LENGTH** | **A** | **G** | **C** | **T** | **Other** |
| --- | --- | --- | --- | --- | --- | --- | --- | --- |
| 1. | NC_031187.1 | *Actinidia tetramera* | 157659 | 48744 | 28579 | 29807 | 50529 | 0 |
| 2. | NC_000932.1 | *Arabidopsis thaliana* | 154478 | 48546 | 27570 | 28496 | 49866 | 0 |
| 3. | MH460639.1 | *Camellia assamica* | 157353 | 48865 | 28831 | 29931 | 49726 | 0 |
| 4. | NC_035574.1 | *Camellia azalea* | 157039 | 48793 | 28708 | 29863 | 49675 | 0 |
| 5. | NC_037472.1 | *Camellia chekiangoleosa* | 156971 | 48761 | 28683 | 29849 | 49678 | 0 |
| 6. | NC_022459.1 | *Camellia cuspidata* | 156618 | 48634 | 28639 | 29800 | 49545 | 0 |
| 7. | NC_022460.1 | *Camellia danzaiensis* | 156576 | 48611 | 28655 | 29810 | 49500 | 0 |
| 8. | NC_024659.1 | *Camellia grandibracteata* | 157127 | 48817 | 28717 | 29878 | 49715 | 0 |
| 9. | NC_022461.1 | *Camellia impressinervis* | 156892 | 48714 | 28703 | 29861 | 49614 | 0 |
| 10. | NC_036830.1 | *Camellia japonica* | 156606 | 48633 | 28639 | 29802 | 49532 | 0 |
| 11. | NC_024660.1 | *Camellia leptophylla* | 157102 | 48807 | 28729 | 29869 | 49697 | 0 |
| 12. | NC_024661.1 | *Camellia petelotii* | 157121 | 48817 | 28721 | 29870 | 49713 | 0 |
| 13. | NC_022462.1 | *Camellia pitardii* | 156585 | 48620 | 28655 | 29807 | 49503 | 0 |
| 14. | NC_024662.1 | *Camellia pubicosta* | 157076 | 48797 | 28708 | 29885 | 49686 | 0 |
| 15. | NC_024663.1 | *Camellia reticulata* | 156971 | 48762 | 28710 | 29853 | 49646 | 0 |
| 16. | NC_020019.1 | *Camellia sinensis* | 157103 | 48813 | 28731 | 29878 | 49680 | 1 |
| 17. | MH019307.1 | *Camellia sinensis* var. *assamica* | 157100 | 48802 | 28711 | 29872 | 49715 | 0 |
| 18. | NC_022264.1 | *Camellia taliensis* | 156974 | 48766 | 28718 | 29864 | 49626 | 0 |
| 19. | NC_022463.1 | *Camellia yunnanensis* | 156592 | 48650 | 28651 | 29797 | 49494 | 0 |
| 20. | MK862266.1 | *Coffea arabica* | 155187 | 47994 | 28507 | 29584 | 49102 | 0 |
| 21. | NC_001319.1 | *Marchantia paleacea* | 121024 | 42896 | 17556 | 17309 | 43263 | 0 |
| 22. | NC_008155.1 | *Oryza sativa* | 134496 | 41231 | 26323 | 26129 | 40813 | 0 |
| 23. | NC_007898.3 | *Solanum lycopersicum* | 155461 | 47640 | 28936 | 29924 | 48961 | 0 |
| 24. | NC_019616.1 | *Vaccinium macrocarpon* | 176045 | 54836 | 31859 | 32930 | 56420 | 0 |
| 25. | NC_007957.1 | *Vitis vinifera* | 160928 | 49793 | 29503 | 30681 | 50951 | 0 |

Table S2. Basic features in shortlisted 7 cp genomes

| Features | *C. assamica* | *C. leptophylla* | *C. sinensis* | *C. pubicosta* | *C. grandibracteata* | *C. sinensis* var. *assamica* | *C. taliensis* |
| --- | --- | --- | --- | --- | --- | --- | --- |
| cp Genome size (bp) | 157353 | 157102 | 157103 | 157076 | 157127 | 157100 | 156974 |
| LSC Region (bp) | 87213 | 86648 | 86645 | 86650 | 86657 | 86649 | 86672 |
| SSC Region (bp) | 18078 | 18276 | 18276 | 18280 | 18286 | 18285 | 18436 |
| IR Region (bp) | 26031 | 26089 | 26091 | 26073 | 26092 | 26083 | 25933 |
| Number of Genes | 126 | 135 | 134 | 135 | 135 | 130 | 137 |
| Protein-coding genes | 89 | 90 | 89 | 90 | 90 | 86 | 89 |
| tRNAs | 29 | 37 | 37 | 37 | 37 | 36 | 40 |
| rRNAs | 8 | 8 | 8 | 8 | 8 | 8 | 8 |

Table S3. List of genes in shortlisted 7 cp genomes

| *C. assamica* | *C. leptophylla* | *C.*  *sinensis* | *C.*  *pubicosta* | *C. grandibracteata* | *C. sinensis* var. *assamica* | *C. taliensis* |
| --- | --- | --- | --- | --- | --- | --- |
| accD | accD | accD | accD | accD | accD | accD |
| atpA | atpA | atpA | atpA | atpA | atpA | atpA |
| atpB | atpB | atpB | atpB | atpB | atpB | atpB |
| atpE | atpE | atpE | atpE | atpE | atpE | atpE |
| atpF | atpF | atpF | atpF | atpF | atpF | atpF |
| atpH | atpH | atpH | atpH | atpH | atpH | atpH |
| atpI | atpI | atpI | atpI | atpI | atpI | atpI |
| ccsA | ccsA | ccsA | ccsA | ccsA | ccsA | ccsA |
| cemA | cemA | cemA | cemA | cemA | cemA | cemA |
| clpP | clpP | clpP | clpP | clpP | clpP | clpP |
| infA | infA | infA | infA | infA | infA | infA |
| matK | matK | matK | matK | matK | matK | matK |
| ndhA | ndhA | ndhA | ndhA | ndhA | ndhA | ndhA |
| ndhB | ndhB | ndhB | ndhB | ndhB | ndhB | ndhB |
| ndhB | ndhB | ndhB | ndhB | ndhB | ndhB | ndhB |
| ndhC | ndhC | ndhC | ndhC | ndhC | ndhC | ndhC |
| ndhD | ndhD | ndhD | ndhD | ndhD | ndhD | ndhD |
| ndhE | ndhE | ndhE | ndhE | ndhE | ndhE | ndhE |
| ndhF | ndhF | ndhF | ndhF | ndhF | ndhF | ndhF |
| ndhG | ndhG | ndhG | ndhG | ndhG | ndhG | ndhG |
| ndhH | ndhH | ndhH | ndhH | ndhH | ndhH | ndhH |
| ndhI | ndhI | ndhI | ndhI | ndhI | ndhI | ndhI |
| ndhJ | ndhJ | ndhJ | ndhJ | ndhJ | ndhJ | ndhJ |
| ndhK | ndhK | ndhK | ndhK | ndhK | ndhK | ndhK |
| petA | petA | petA | petA | petA | petA | petA |
| petB | petB | petB | petB | petB | petB | petB |
| petD | petD | petD | petD | petD | petD | petD |
| petG | petG | petG | petG | petG | petG | petG |
| petL | petL | petL | petL | petL | petL | petL |
| petN | petN | petN | petN | petN | petN | petN |
| psaA | psaA | psaA | psaA | psaA | psaA | psaA |
| psaB | psaB | psaB | psaB | psaB | psaB | psaB |
| psaC | psaC | psaC | psaC | psaC | psaC | psaC |
| psaI | psaI | psaI | psaI | psaI | psaI | psaI |
| psaJ | psaJ | psaJ | psaJ | psaJ | psaJ | psaJ |
| psbA | psbA | psbA | psbA | psbA | psbA | psbA |
| psbB | psbB | psbB | psbB | psbB | psbB | psbB |
| psbC | psbC | psbC | psbC | psbC | psbC | psbC |
| psbD | psbD | psbD | psbD | psbD | psbD | psbD |
| psbE | psbE | psbE | psbE | psbE | psbE | psbE |
| psbF | psbF | psbF | psbF | psbF | psbF | psbF |
| psbH | psbH | psbH | psbH | psbH | psbH | psbH |
| psbI | psbI | psbI | psbI | psbI | psbI | psbI |
| psbJ | psbJ | psbJ | psbJ | psbJ | psbJ | psbJ |
| psbK | psbK | psbK | psbK | psbK | psbK | psbK |
| psbL | psbL | psbL | psbL | psbL | psbL | psbL |
| psbM | psbM | psbM | psbM | psbM | psbM | psbM |
| psbN | psbN | psbN | psbN | psbN | psbN | psbN |
| psbT | psbT | psbT | psbT | psbT | psbT | psbT |
| psbZ | psbZ | psbZ | psbZ | psbZ | psbZ | --- |
| rbcL | rbcL | rbcL | rbcL | rbcL | rbcL | rbcL |
| rpl14 | rpl14 | rpl14 | rpl14 | rpl14 | rpl14 | rpl14 |
| rpl16 | rpl16 | rpl16 | rpl16 | rpl16 | rpl16 | rpl16 |
| rpl2 | rpl2 | rpl2 | rpl2 | rpl2 | rpl2 | rpl2 |
| rpl2 | rpl2 | rpl2 | rpl2 | rpl2 | rpl2 | rpl2 |
| rpl20 | rpl20 | rpl20 | rpl20 | rpl20 | rpl20 | rpl20 |
| rpl22 | rpl22 | rpl22 | rpl22 | rpl22 | rpl22 | rpl22 |
| rpl23 | rpl23 | rpl23 | rpl23 | rpl23 | rpl23 | rpl23 |
| rpl23 | rpl23 | rpl23 | rpl23 | rpl23 | rpl23 | rpl23 |
| rpl32 | rpl32 | rpl32 | rpl32 | rpl32 | rpl32 | rpl32 |
| rpl33 | rpl33 | rpl33 | rpl33 | rpl33 | rpl33 | rpl33 |
| rpl36 | rpl36 | rpl36 | rpl36 | rpl36 | rpl36 | rpl36 |
| rpoA | rpoA | rpoA | rpoA | rpoA | rpoA | rpoA |
| rpoB | rpoB | rpoB | rpoB | rpoB | rpoB | rpoB |
| rpoC1 | rpoC1 | rpoC1 | rpoC1 | rpoC1 | rpoC1 | rpoC1 |
| rpoC2 | rpoC2 | rpoC2 | rpoC2 | rpoC2 | rpoC2 | rpoC2 |
| rps11 | rps11 | rps11 | rps11 | rps11 | rps11 | rps11 |
| rps12 | rps12 | rps12 | rps12 | rps12 | rps12 | rps12 |
| rps12 | rps12 | rps12 | rps12 | rps12 | --- | rps12 |
| rps14 | rps14 | rps14 | rps14 | rps14 | rps14 | rps14 |
| rps15 | rps15 | rps15 | rps15 | rps15 | rps15 | rps15 |
| rps16 | rps16 | rps16 | rps16 | rps16 | rps16 | rps16 |
| rps18 | rps18 | rps18 | rps18 | rps18 | rps18 | rps18 |
| rps19 | rps19 | rps19 | rps19 | rps19 | --- | rps19 |
| --- | --- | rps19 | --- | --- | --- | --- |
| rps2 | rps2 | rps2 | rps2 | rps2 | rps2 | rps2 |
| rps3 | rps3 | rps3 | rps3 | rps3 | rps3 | rps3 |
| rps4 | rps4 | rps4 | rps4 | rps4 | rps4 | rps4 |
| rps7 | rps7 | rps7 | rps7 | rps7 | rps7 | rps7 |
| rps7 | rps7 | rps7 | rps7 | rps7 | rps7 | rps7 |
| rps8 | rps8 | rps8 | rps8 | rps8 | rps8 | rps8 |
| ycf1 | ycf1 | ycf1 | ycf1 | ycf1 | ycf1 | ycf1 |
| ycf15 | ycf1 | ycf1 | ycf1 | ycf1 | ycf1 | ycf15 |
| ycf15 | ycf15 | ycf15 | ycf15 | ycf15 | ycf15 | ycf15 |
| ycf15 | ycf15 | ycf15 | ycf15 | ycf15 | ycf15 | ycf2 |
| ycf15 | ycf2 | ycf2 | ycf2 | ycf2 | ycf2 | ycf2 |
| ycf2 | ycf2 | ycf2 | ycf2 | ycf2 | ycf2 | ycf3 |
| ycf2 | ycf3 | ycf3 | ycf3 | ycf3 | ycf3 | ycf4 |
| ycf3 | ycf4 | ycf4 | ycf4 | ycf4 | ycf4 | orf188 |
| ycf4 | orf42 | rrn16 | orf42 | orf42 | rrn16S | orf42 |
| rrn16S | orf42 | rrn16 | orf42 | orf42 | rrn16S | lhbA |
| rrn16S | rrn16S | rrn23 | rrn16S | rrn16S | rrn23S | rrn16 |
| rrn23S | rrn16S | rrn23 | rrn16S | rrn16S | rrn23S | rrn16 |
| rrn23S | rrn23S | rrn4.5 | rrn23S | rrn23S | rrn4.5S | rrn23 |
| rrn4.5S | rrn23S | rrn4.5 | rrn23S | rrn23S | rrn4.5S | rrn23 |
| rrn4.5S | rrn4.5S | rrn5 | rrn4.5S | rrn4.5S | rrn5S | rrn4.5 |
| rrn5S | rrn4.5S | rrn5 | rrn4.5S | rrn4.5S | rrn5S | rrn4.5 |
| rrn5S | rrn5S | tRNA-Ala | rrn5S | rrn5S | trnA-UGC | rrn5 |
| trnC-GCA | rrn5S | tRNA-Ala | rrn5S | rrn5S | trnC-GCA | rrn5 |
| trnD-GTC | trnA-UGC | tRNA-Arg | trnA-UGC | trnA-UGC | trnD-GUC | trnA-UGC |
| trnE-TTC | trnA-UGC | tRNA-Arg | trnA-UGC | trnA-UGC | trnE-UUC | trnA-UGC |
| trnF-GAA | trnC-GCA | tRNA-Arg | trnC-GCA | trnC-GCA | trnF-GAA | trnC-GCA |
| trnG-GCC | trnD-GUC | tRNA-Asn | trnD-GUC | trnD-GUC | trnfM-CAU | trnD-GUC |
| trnH-GTG | trnE-UUC | tRNA-Asn | trnE-UUC | trnE-UUC | trnG-UCC | trnE-UUC |
| trnI-CAT | trnF-GAA | tRNA-Asp | trnF-GAA | trnF-GAA | trnG-UCC | trnF-GAA |
| trnI-CAT | trnfM-CAU | tRNA-Cys | trnfM-CAU | trnfM-CAU | trnH-GUG | trnfM-CAU |
| trnL-CAA | trnG-GCC | tRNA-fM | trnG-GCC | trnG-GCC | trnI-CAU | trnG |
| trnL-CAA | trnG-UCC | tRNA-Gln | trnG-UCC | trnG-UCC | trnI-CAU | trnG-UCC |
| trnL-TAG | trnH-GUG | tRNA-Glu | trnH-GUG | trnH-GUG | trnI-GAU | trnH-GUG |
| trnM-CAT | trnI-CAU | tRNA-Gly | trnI-CAU | trnI-CAU | trnI-GAU | trnI-CAU |
| trnM-CAT | trnI-CAU | tRNA-Gly | trnI-CAU | trnI-CAU | trnK-UUU | trnI-CAU |
| trnN-GTT | trnI-GAU | tRNA-His | trnI-GAU | trnI-GAU | trnL-CAA | trnI-GAU |
| trnN-GTT | trnI-GAU | tRNA-His | trnI-GAU | trnI-GAU | trnL-CAA | trnI-GAU |
| trnP-TGG | trnK-UUU | tRNA-His | trnK-UUU | trnK-UUU | trnL-UAA | trnK-UUU |
| trnQ-TTG | trnL-CAA | tRNA-Ile | trnL-CAA | trnL-CAA | trnL-UAG | trnL-CAA |
| trnR-ACG | trnL-CAA | tRNA-Ile | trnL-CAA | trnL-CAA | trnM-CAU | trnL-CAA |
| trnR-ACG | trnL-UAA | tRNA-Leu | trnL-UAA | trnL-UAA | trnN-GUU | trnL-UAA |
| trnR-TCT | trnL-UAG | tRNA-Leu | trnL-UAG | trnL-UAG | trnN-GUU | trnL-UAG |
| trnS-GCT | trnM-CAU | tRNA-Leu | trnM-CAU | trnM-CAU | trnP-UGG | trnM-CAU |
| trnS-GGA | trnN-GUU | tRNA-Leu | trnN-GUU | trnN-GUU | trnQ-UUG | trnM-CAU |
| trnS-TGA | trnN-GUU | tRNA-Lys | trnN-GUU | trnN-GUU | trnR-ACG | trnN-GUU |
| trnT-GGT | trnP-UGG | tRNA-Met | trnP-UGG | trnP-UGG | trnR-ACG | trnN-GUU |
| trnT-TGT | trnQ-UUG | tRNA-Phe | trnQ-UUG | trnQ-UUG | trnR-UCU | trnP-GGG |
| trnV-GAC | trnR-ACG | tRNA-Pro | trnR-ACG | trnR-ACG | trnS-GCU | trnP-UGG |
| trnV-GAC | trnR-ACG | tRNA-Ser | trnR-ACG | trnR-ACG | trnS-GGA | trnQ-UUG |
| trnW-CCA | trnR-UCU | tRNA-Ser | trnR-UCU | trnR-UCU | trnS-UGA | trnR-ACG |
| trnY-GTA | trnS-GCU | tRNA-Ser | trnS-GCU | trnS-GCU | trnT-GGU | trnR-ACG |
| --- | trnS-GGA | tRNA-Thr | trnS-GGA | trnS-GGA | trnT-UGU | trnR-UCU |
| --- | trnS-UGA | tRNA-Thr | trnS-UGA | trnS-UGA | trnV-GAC | trnS-GCU |
| --- | trnT-GGU | tRNA-Trp | trnT-GGU | trnT-GGU | trnV-GAC | trnS-GGA |
| --- | trnT-UGU | tRNA-Tyr | trnT-UGU | trnT-UGU | trnV-UAC | trnS-UGA |
| --- | trnV-GAC | tRNA-Val | trnV-GAC | trnV-GAC | trnW-CCA | trnT-GGU |
| --- | trnV-GAC | tRNA-Val | trnV-GAC | trnV-GAC | trnY-GUA | trnT-GGU |
| --- | trnV-UAC | tRNA-Val | trnV-UAC | trnV-UAC | --- | trnT-UGU |
| --- | trnW-CCA | --- | trnW-CCA | trnW-CCA | --- | trnV-GAC |
| --- | trnY-GUA | --- | trnY-GUA | trnY-GUA | --- | trnV-GAC |
| --- | --- | --- | --- | --- | --- | trnV-UAC |
| --- | --- | --- | --- | --- | --- | trnW-CCA |
| --- | --- | --- | --- | --- | --- | trnY-GUA |

Table S4. Distribution to different repeat type classes

| SSR Motif Type | *C. assamica* | *C. leptophylla* | *C. sinensis* | *C. pubicosta* | *C. grandibracteata* | *C. sinensis* var. *assamica* | *C. taliensis* |
| --- | --- | --- | --- | --- | --- | --- | --- |
| Mono | 157 | 157 | 157 | 156 | 159 | 160 | 158 |
| Di | 41 | 41 | 41 | 41 | 41 | 41 | 41 |
| Tri | 1 | 1 | 1 | 1 | 1 | 1 | 2 |
| Tetra | 11 | 12 | 11 | 11 | 10 | 10 | 10 |
| Hexa | 2 | 2 | 2 | 2 | 2 | 2 | 0 |
| Total | 212 | 213 | 212 | 211 | 213 | 214 | 211 |

Table S5. Frequency of identified SSR motifs

| SSR motifs | *C. assamica* | *C. leptophylla* | *C. sinensis* | *C. pubicosta* | *C. grandibracteata* | *C. sinensis* var. *assamica* | *C. taliensis* |
| --- | --- | --- | --- | --- | --- | --- | --- |
| A | 61 | 63 | 64 | 62 | 61 | 61 | 64 |
| C | 3 | 3 | 3 | 3 | 3 | 3 | 3 |
| G | 2 | 2 | 2 | 2 | 2 | 2 | 2 |
| T | 91 | 89 | 88 | 89 | 93 | 94 | 89 |
| AG | 3 | 3 | 3 | 3 | 3 | 3 | 3 |
| AT | 15 | 15 | 15 | 15 | 15 | 15 | 15 |
| CT | 3 | 3 | 3 | 3 | 3 | 3 | 3 |
| GA | 5 | 5 | 5 | 5 | 5 | 5 | 5 |
| TA | 10 | 10 | 10 | 10 | 10 | 10 | 10 |
| TC | 5 | 5 | 5 | 5 | 5 | 5 | 5 |
| ATT | 0 | 0 | 0 | 0 | 0 | 0 | 1 |
| TTC | 1 | 1 | 1 | 1 | 1 | 1 | 1 |
| AAAT | 2 | 2 | 2 | 2 | 2 | 2 | 0 |
| AATA | 0 | 1 | 1 | 1 | 1 | 1 | 1 |
| AGAT | 1 | 1 | 1 | 1 | 1 | 1 | 1 |
| ATAG | 1 | 1 | 1 | 1 | 1 | 1 | 1 |
| CCCT | 1 | 1 | 1 | 1 | 0 | 0 | 1 |
| GAAA | 1 | 1 | 1 | 1 | 1 | 1 | 1 |
| GAGG | 1 | 1 | 1 | 0 | 0 | 0 | 1 |
| GTCT | 1 | 1 | 1 | 1 | 1 | 1 | 1 |
| TCTA | 1 | 1 | 1 | 1 | 1 | 1 | 1 |
| TCTT | 1 | 1 | 0 | 1 | 1 | 1 | 1 |
| TTTC | 1 | 1 | 1 | 1 | 1 | 1 | 1 |
| AAAAAG | 1 | 1 | 1 | 1 | 1 | 1 | 0 |
| CTTTTT | 1 | 1 | 1 | 1 | 1 | 1 | 0 |

Table S6. The codon usage frequency distribution in terms of RSCU values among seven cp genomes Camellia genus

| Amino Acid | Codon | *C. assamica* | *C. leptophylla* | *C. sinensis* | *C. pubicosta* | *C. grandibracteata* | *C. sinensis* var. *assamica* | *C. taliensis* |
| --- | --- | --- | --- | --- | --- | --- | --- | --- |
| A | GCA | 1.112 | 1.145 | 1.137 | 1.142 | 1.14 | 1.137 | 1.145 |
| A | GCC | 0.66 | 0.64 | 0.647 | 0.64 | 0.64 | 0.642 | 0.648 |
| A | GCG | 0.393 | 0.395 | 0.386 | 0.392 | 0.392 | 0.383 | 0.38 |
| A | GCT | 1.834 | 1.821 | 1.83 | 1.827 | 1.828 | 1.839 | 1.827 |
| C | TGC | 0.488 | 0.468 | 0.468 | 0.468 | 0.468 | 0.475 | 0.466 |
| C | TGT | 1.512 | 1.532 | 1.532 | 1.532 | 1.532 | 1.525 | 1.534 |
| D | GAC | 0.381 | 0.37 | 0.366 | 0.369 | 0.369 | 0.369 | 0.361 |
| D | GAT | 1.619 | 1.63 | 1.634 | 1.631 | 1.631 | 1.631 | 1.639 |
| E | GAA | 1.486 | 1.527 | 1.512 | 1.528 | 1.529 | 1.508 | 1.509 |
| E | GAG | 0.514 | 0.473 | 0.488 | 0.472 | 0.471 | 0.492 | 0.491 |
| F | TTC | 0.733 | 0.68 | 0.713 | 0.68 | 0.684 | 0.723 | 0.72 |
| F | TTT | 1.267 | 1.32 | 1.287 | 1.32 | 1.316 | 1.277 | 1.28 |
| G | GGA | 1.625 | 1.633 | 1.641 | 1.633 | 1.634 | 1.637 | 1.648 |
| G | GGC | 0.415 | 0.42 | 0.417 | 0.418 | 0.42 | 0.413 | 0.41 |
| G | GGG | 0.679 | 0.651 | 0.676 | 0.651 | 0.649 | 0.674 | 0.673 |
| G | GGT | 1.281 | 1.296 | 1.266 | 1.298 | 1.297 | 1.276 | 1.269 |
| H | CAC | 0.44 | 0.415 | 0.425 | 0.417 | 0.419 | 0.432 | 0.426 |
| H | CAT | 1.56 | 1.585 | 1.575 | 1.583 | 1.581 | 1.568 | 1.574 |
| I | ATA | 0.931 | 0.959 | 0.963 | 0.957 | 0.955 | 0.958 | 0.959 |
| I | ATC | 0.609 | 0.585 | 0.596 | 0.586 | 0.583 | 0.599 | 0.6 |
| I | ATT | 1.46 | 1.456 | 1.441 | 1.457 | 1.462 | 1.442 | 1.441 |
| K | AAA | 1.463 | 1.519 | 1.487 | 1.521 | 1.521 | 1.481 | 1.492 |
| K | AAG | 0.537 | 0.481 | 0.513 | 0.479 | 0.479 | 0.519 | 0.508 |
| L | CTA | 0.814 | 0.799 | 0.799 | 0.802 | 0.803 | 0.806 | 0.813 |
| L | CTC | 0.464 | 0.419 | 0.432 | 0.421 | 0.421 | 0.446 | 0.429 |
| L | CTG | 0.385 | 0.373 | 0.393 | 0.374 | 0.373 | 0.394 | 0.393 |
| L | CTT | 1.248 | 1.235 | 1.254 | 1.233 | 1.238 | 1.26 | 1.266 |
| L | TTA | 1.849 | 1.984 | 1.905 | 1.98 | 1.981 | 1.889 | 1.896 |
| L | TTG | 1.241 | 1.189 | 1.217 | 1.19 | 1.185 | 1.204 | 1.202 |
| N | AAC | 0.469 | 0.446 | 0.457 | 0.45 | 0.448 | 0.458 | 0.463 |
| N | AAT | 1.531 | 1.554 | 1.543 | 1.55 | 1.552 | 1.542 | 1.537 |
| P | CCA | 1.176 | 1.186 | 1.191 | 1.182 | 1.181 | 1.176 | 1.187 |
| P | CCC | 0.702 | 0.695 | 0.704 | 0.699 | 0.696 | 0.703 | 0.698 |
| P | CCG | 0.496 | 0.488 | 0.502 | 0.488 | 0.489 | 0.509 | 0.507 |
| P | CCT | 1.626 | 1.631 | 1.603 | 1.631 | 1.634 | 1.612 | 1.609 |
| Q | CAA | 1.511 | 1.539 | 1.525 | 1.539 | 1.539 | 1.528 | 1.534 |
| Q | CAG | 0.489 | 0.461 | 0.475 | 0.461 | 0.461 | 0.472 | 0.466 |
| R | AGA | 1.783 | 1.855 | 1.856 | 1.855 | 1.854 | 1.862 | 1.864 |
| R | AGG | 0.628 | 0.563 | 0.621 | 0.572 | 0.573 | 0.624 | 0.614 |
| R | CGA | 1.451 | 1.456 | 1.442 | 1.452 | 1.455 | 1.441 | 1.432 |
| R | CGC | 0.344 | 0.329 | 0.331 | 0.329 | 0.326 | 0.331 | 0.324 |
| R | CGG | 0.428 | 0.391 | 0.422 | 0.391 | 0.391 | 0.421 | 0.417 |
| R | CGT | 1.367 | 1.406 | 1.329 | 1.402 | 1.401 | 1.32 | 1.35 |
| S | AGC | 0.346 | 0.348 | 0.343 | 0.348 | 0.348 | 0.338 | 0.337 |
| S | AGT | 1.249 | 1.267 | 1.215 | 1.266 | 1.266 | 1.229 | 1.227 |
| S | TCA | 1.141 | 1.182 | 1.191 | 1.181 | 1.181 | 1.192 | 1.187 |
| S | TCC | 0.959 | 0.899 | 0.941 | 0.899 | 0.899 | 0.937 | 0.947 |
| S | TCG | 0.515 | 0.476 | 0.508 | 0.479 | 0.479 | 0.521 | 0.512 |
| S | TCT | 1.789 | 1.828 | 1.801 | 1.827 | 1.827 | 1.782 | 1.79 |
| T | ACA | 1.21 | 1.222 | 1.229 | 1.227 | 1.227 | 1.235 | 1.217 |
| T | ACC | 0.728 | 0.745 | 0.73 | 0.746 | 0.744 | 0.732 | 0.732 |
| T | ACG | 0.417 | 0.384 | 0.412 | 0.384 | 0.383 | 0.419 | 0.416 |
| T | ACT | 1.644 | 1.65 | 1.629 | 1.643 | 1.646 | 1.614 | 1.635 |
| V | GTA | 1.491 | 1.521 | 1.503 | 1.526 | 1.527 | 1.493 | 1.501 |
| V | GTC | 0.474 | 0.456 | 0.462 | 0.452 | 0.453 | 0.477 | 0.467 |
| V | GTG | 0.558 | 0.534 | 0.555 | 0.535 | 0.537 | 0.553 | 0.551 |
| V | GTT | 1.477 | 1.488 | 1.481 | 1.487 | 1.482 | 1.476 | 1.481 |
| W | TGG | 1 | 1 | 1 | 1 | 1 | 1 | 1 |
| Y | TAC | 0.377 | 0.401 | 0.395 | 0.4 | 0.4 | 0.388 | 0.392 |
| Y | TAT | 1.623 | 1.599 | 1.605 | 1.6 | 1.6 | 1.612 | 1.608 |
